# The Suitability of Potential Organ Donors Using Real Case-Scenarios; Do we Need to Create a “Donor Board” Process for Donors Perceived as Unlikely Suitable?

**DOI:** 10.3389/ti.2022.10107

**Published:** 2022-03-10

**Authors:** Pierre Marsolais, Gabrielle Larouche, Anne-Marie Lagacé, Virginie Williams, Karim Serri, Francis Bernard, Philippe Rico, Anne Julie Frenette, David Williamson, Martin Albert, Emmanuel Charbonney

**Affiliations:** ^1^ Hôpital du Sacré-Coeur de Montréal (CIUSSS-NIM), Montréal, QC, Canada; ^2^ Faculty of Medicine, Université de Montréal, Montréal, QC, Canada; ^3^ Faculty of Medecine, Université de Sherbrooke, Sherbrooke, QC, Canada; ^4^ Facutly of Pharmacy, Université de Montréal, Montréal, QC, Canada; ^5^ Centre Hospitalier de l’Université de Montréal, Montréal, QC, Canada

**Keywords:** organ procurement, potential donors, transplant, critical care, system

## Abstract

**Introduction:** Despite availability of selection criteria, different interpretations can lead to variability in the appreciation of donor eligibility with possible viable organs missed. Our primary objective was to test the perception of feasibility of potential organ donors through the survey of a small sample of external evaluators.

**Methods:** Clinical scenarios summarizing 66 potential donors managed in the first year of our Organ Recovery Center were sent to four critical care physicians to evaluate the feasibility of the potential donors and the probability of organ procurement.

**Results:** Potential donors procuring at least one organ were identified in 55 of the 66 cases (83%). Unanimity was reached in 38 cases, encompassing 35 out of the 55 converted and 3 of the non-converted donors. The overall agreement was moderate (kappa = 0.60, 95% CI: 0.37–0.82). For the organs finally procured for transplantation, organ donation was predicted for the majority of the cases, but high discrepancy was present with the final outcome of organs not procured (particularly liver and kidney).

**Conclusion:** The assessment of a potential donor is a complex dynamic process. In order to increase organ availability, standardized electronically clinical data, as well a “donor board” structure of decision might inform future systems.

## Graphical Abstract

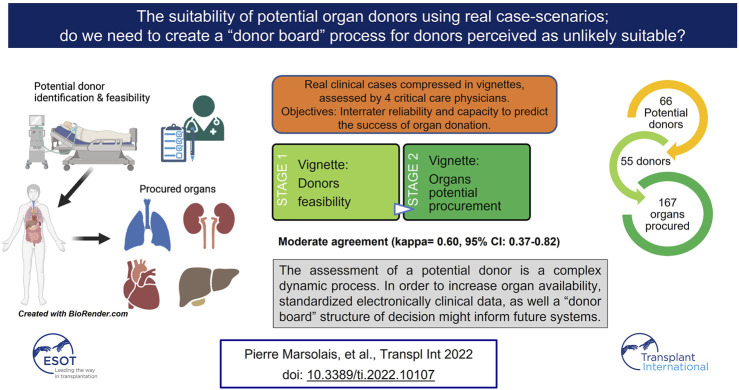

## Introduction

Worldwide organ shortage is a major issue in the field of organ transplantation (1, 2). In 2018, with 20.6 donors by million population, a total of 2,782 transplants were performed while 223 people died waiting for an organ in Canada (3). In recent years, numerous initiatives have improved the yield of organ donation, including campaigns targeting the adherence of the general population to organ donation, promotion of presumed consent and living donation, guidelines for donor management (4, 5), organisational structure (6, 7), introduction of liver splits, reintroduction of Donation after Cardiac Death (DCD) (8, 9) and amongst others, extended criteria (10). Despite these significant efforts, the gap between the number of organs offered and the demand remains.

The critical care physician is a central stakeholder in the optimization of organs, while the entire process leading to organ donation from an identified potential donor relies often on numerous caregivers, including the Organ Procurement Organization’s (OPO) personal and the transplantation programs (11). The identification of donors, medical staff attitudes and institutional culture have been identified as sources of missed opportunities (12, 13). Unfortunately, despite the presence of selection criteria, different interpretations can lead to important variability in the appreciation of the eligibility of a donor, with many viable organs missed (14, 15). Several studies have reported cases of organs being first refused by an institution and then successfully transplanted after acceptation by another (16–18), reflecting the variability in acceptance on the transplant team side. Even if the relation between success of donation, communication of donor information and processes of decision making seems important, it may be difficult to isolate specific culprits in a complex and fragmented system.

Preliminary reports suggest the impact of a dedicated team on organ donation, applying organ management to increase the conversion of patients into donors (19, 20). However, little is known regarding the supporting donor team, particularly regarding how critical care physicians assess potential donors and feasibility of organ donation. As summarized by Tong et al, qualitative studies are required in order to understand the process of decisions, central to the improvement of transplant care (21). The overall objective of the present study was to evaluate the perception of feasibility of a group of potential organ donors, through the survey of a small sample of external evaluators. Our hypothesis was that the blind comparison of the evaluator’s perceptions of feasibility with the outcome of organ donation would allow us to identify barriers or potential directions to improve donor’s realization.

## Materials and Methods

### Sources of Data

The study was conducted in 2016–2017 and retrospectively selected a group of potential donors referred to our Organ Recovery Center (ORC) in its first year of activity (June 2013–June 2014) (19). The study data sources were the medical chart, scanned electronic documents, resource nurse’s donor files, and the provincial OPO coordinator dataset. Laboratory, radiology and investigations (i.e., bronchoscopy, echocardiography, pathology) results were collected. The study was approved by the institutional research ethic board (CER 2014–1049).

### Population and Pre-Defined Level of Donors’ Potentiality

All potential donation after brain death (DBD) and DCD admitted during the first year of our ORC activity, either directly from our ICU or transferred from other centers, were included. As for any case entered into the database, they were categorized into 2 categories: 1) those who had no obvious problem to be converted (feasible donors) because they completely matched the local OPO criteria and requirements; 2) those who had been identified by the ORC as unlikely to be converted (a priori unfeasible) after the initial assessment but still supported. The latter were identified in regard to either OPO guidelines, suspicion of neoplasia or identified legal/administrative barriers. This assessment was done *a priori* according to the perception of the ORC team and collected systematically at admission. As for the definite outcome of donation for transplant, two sub-categories were defined, namely converted (at least one organ transplanted) vs none converted donors.

### Clinical Case Scenario Vignettes Development

Using the collected information from the 66 potential donors, we developed clinical vignettes in the form of clinical case-scenarios (22). The presented information was anonymized and standardized to protect the privacy of patients. The vignettes were built in two parts (example in Supplementary Material): the first page contained a short description of the potential donor at the time of admission in the ORC (if transferred) or at the time of consent for organ donation in our center. The second page described the clinical information, radiological assessments and the physiological evolution of the following organs: heart, lungs, kidneys and liver, excluding the pancreas. We did not consider the pancreas because of the very restrictive criteria for this organ based on the age below 50 years, Body mass index under 30 and the absence of diabetes.

The content of each vignette was examined by the investigators, who reviewed the clarity and comprehensiveness of items, individually or in group, until an agreement was reached regarding the format and content. Two internal evaluators (intensivists working in our center), who were not part of the study, were sent a random sample of 10 vignettes, to assess the format and the content, comprehensiveness, clarity or the inaccuracy of information. Based on their comments, controls of information extracted from the patient’s file for all the vignettes were made, as well as complements or modifications suggested after internal review. Modification of the format and items display were made according to their feedback.

### Statistics

The sample size calculation was based on a kappa null value set at 0.4 and an expected significant difference to be 0.2, with a kappa of 0.6 for reached agreement. Considering an expected proportion of mean positive rating at 0.7 (to the question of feasible candidate or not) and power of 80%, the number of comparisons needed were 191. Of the 200 comparisons (4 × 50 vignettes), agreement testing was analyzed using Gwet kappa coefficient and the level of agreement scaled (23). Results were reported using descriptive statistics as proportions of categorical or ordinal variables and kappa were reported with 95% confidence intervals. A Fisher’s exact test was used to compare proportions. *p* Value was deemed significant if < 0.05. Statistics were processed using IBM SPSS 20.

### Design of Rating and Assessment Processing

The 66 vignettes were evaluated, with four blocks of a random sample of 50 vignettes sent to 4 critical care physicians from centers outside of the ORC service corridor. We aimed to establish the interrater reliability as primary objective and their capacity to predict donation outcome as the secondary objective. Each vignette was then evaluated by three physicians, except for two vignettes evaluated by the four physicians (Total sample of 50 × 4 = 200; 66 vignettes × 3 = 198). These physicians were involved in organ donor management on a regular basis and affiliated to the 4 hospitals with the highest volume of Quebec OPO referral. We capped the evaluation at 50 vignettes, to maximize their adherence to the process (4 × 50 = 200 assessments) and according to the sample size calculation. Initially, we sent the first page (Supplementary Material) detailing the general description of the patient after consent for organ donation. Clinicians were asked to state if they thought the potential donor presented on the vignette was a feasible organ donor, within the framework of OPO guidelines. They received the instruction to give an answer based only on the information available, their knowledge, their judgement and their usual work environment. If they answered *yes*, they had to rate the probability of the organ donation outcome, as low, medium or high. After returning their answer, they were sent the second page with organs data, only for the cases they had deemed feasible. Based on the description, the clinicians rated their perception of suitability for transplantation (Supplementary Material); if they thought that it was the case, they had to rate the likelihood on an ordinal scale of categorical percentages (<20%, 20–40%, 40–60%, 60–80%, 80–100) for every organ separately. They were not aware, at any time, of the final organ donation outcome.

## Results

### Potential Donors’ Characteristics

During the first year of activity of the ORC, we managed 66 potential donors with a median age of 57 years. The majority of them were referred from other centers (56%) and cerebral hemorrhage was the most frequent brain injury (44%), as shown in Table 1. The number of potential donors converted in organ donors was 55/66 (83%), of which 6/55 (11%) were DCD.

**TABLE 1:** Potential donors’ characteristics.

	N = 66
Age (years), median (range)	57 (17–84)
Female/Male, N	29/37
Deceased neurologically, N (%)	59 (89)
Causes of brain injury, N (%)	
Brain Anoxia	19 (29)
Cerebral Hemorrhage	29 (44)
Ischemic Stroke	3 (4.5)
Brain Trauma	14 (21)
Cerebral tumor	1 (1,5)
PoDo References, N (%)	
From our center	29 (44)
From other centers	37 (56)
Converted Donors, N (%)	55 (83)
Female/Male, N	24/31
Age (years), median (range)	53 (17–84)
DBD, N (%)	49 (89)
DCD, N (%)	6 (11)
Converted Donors from other centers, N (%)	30 (55)

Results are displayed as N (%) or Median (Range).

PoDo, Potential donors; ORC, Organ Recovery Center; DBD, Donation after Brain Death; DCD, Donation after Cardiac Death.

All of the cases deemed feasible after the initial evaluation by the ORC were converted except one, whereas 63% of the cases deemed unfeasible were converted (Table 2). The causes for non-conversion of the potential donors were cancer (N = 3), infection (N = 1), circulatory collapse (N = 1), family withdrawal of consent (N = 1), no suitable organ (N = 3) and age related (N = 1).

**TABLE 2:** *A priory* feasibility according to Organ Recovery Center (ODC).

ORC categories	Converted, N = 55	Not converted, N = 11
A. Feasible, N = 39	38	1
B. *Unfeasible, N = 27	17	10

*Identified in regard to either OPO guidelines, suspicion of neoplasia or identified legal/administrative barriers.

### Rating and Assessment of Potential Donors

Clinicians deemed the potential donors as feasible, for various proportions of the vignettes received (A: 72%, B: 100%, C: 60%, D: 80%). The feasibility rating of potential donors by clinicians is presented in Table 3. Of the 66 vignettes (first part), one case was rated feasible by none, 14 were rated feasible by one, 13 by two, 38 by three or more clinicians. Therefore, unanimity was reached in 38 cases, encompassing 35 out of the 55 converted and 3 of the non-converted donors. The overall agreement, for the same cases assessed, between clinicians was moderate (kappa = 0.60, 95% CI: 0.37–0.82). Three clinicians reported weak feasibility for 2.5–4% of their realistic cases, and one for 27% of them. The aggregation of the weak probability of feasibility category with “not feasible” did not increase their agreement level. The agreement between the converted donors and rating of the clinicians were good for two, moderate for one and fair for one (*Kappa*, Table 3).

**TABLE 3:** Proportion of potential donors rated as feasible by external clinicians.

Organ donation outcome	Feasibility rating	A (N = 50)	B (N = 50)	C (N = 50)	D (N = 50)
Converted donors (N = 55)	N/total (%)	33/40 (82)	41/41 (100)	27/43 (63)	34/42 (81)
Not Converted donors (N = 11)	N/total (%)	3/10 (30)	9/9 (100)	3/7 (43)	6/8 (75)
	Deemed feasible, proportion (%)	36/50 (72)	50/50 (100)	30/50 (60)	40/50 (80)
Kappa* (95% interval)		0.69 (0.51–0.86)	0.78 (0.66–0.91)	0.37 (0.15–0.60)	0.60 (0.42–0.78)
PoDo converted assessed, N/total (%)		40/55 (73)	41/55 (75)	43/55 (78)	42/55 (76)
PoDo not converted assessed, N/total (%)		10/11 (91)	9/11 (82)	7/11 (64)	8/11 (73)

*Agreement *Kappa* between the converted donors and each clinician rating (*p* < 0.0001). The proportion of PoDo converted or not, received for assessment by each clinician are reported in the lower part of the table.

The first column (upper part of table) give the absolute numbers of potential donors (PoDo) converted or not. The proportion of PoDo deemed feasible by each clinician for these two categories are the displayed in the four last columns.

ORC, Organ Recovery Center.

Taking the final outcome as reference, the sensitivity of clinician to predict a converted potential donor was 87%, and specificity 31%. The positive predicted value was 86.5% and negative predictive value was 41%. Regarding the subgroup of predefined unfeasible potential donors, the clinicians (at least one) rated them feasible in more than 50% for those finally converted (median 66.5%; range 36–100%), but less than 50% for those not converted (median 43.5%; range 13–100%). Of the 17 cases deemed initially unfeasible by the ORC team but finally converted, 10 potential donors were deemed feasible by more than one clinician. For the feasible subgroup, their feasibility rate assessment was the highest (median 91%; range 76–100%).

### Perceived Barriers of Converted Potential Donors

The number of converted potential donors assessed by each clinician was very similar (Table 3). Various proportions were deemed not feasible by clinicians (A: 18%, B: 0%, C: 37%, D: 19%; Table 3, first line). Of the 55 converted donors, clinicians deemed 20 cases (36%) not feasible (10 were by one clinician, 9 were by two clinicians and 1 by three clinicians).

The presence of non-admissible criteria according to the OPO and pathology that could be perceived as a barrier were present in the 10 cases, where at least two clinicians had declined feasibility. Despite the opportunity and request to describe a reason for non-feasibility, only four cases had comments written by the clinicians. All of the 10 potential donors had multiple organ failure at the time of support initiation (circulatory shock, acute renal failure, shock liver, coagulopathy or high lactate). Two cases were in a situation where the coroner was involved. One had a 9 mm suspicious lung nodule and another had multiple suspicious mediastinal adenopathies on CT-scan. Four of the potential donors had an aspiration pneumonia with significant lung infiltrates, with one of them also having an urosepsis; another had an endocarditis with cerebral embolization. Finally, one patient had trisomy and one had an HIV positive screening test (false positivity revealed later).

### Potential Donors Deemed Feasible by Clinicians but not Converted

We found 6 potential donors deemed feasible by at least two clinicians, but not converted. One had positive Hepatitis C, with no suitable receiver once offered through the OPO; one had a highly suspected renal carcinoma and two others had non-resolving multiple organ failure with refractory shock. One transferred patient failed the criteria for DBD after assessment by the ORC team and was not suitable for DCD. Finally, consent was withdrawn by the family of a last one.

### Organ Feasibility Rating

**FIGURE 1:**
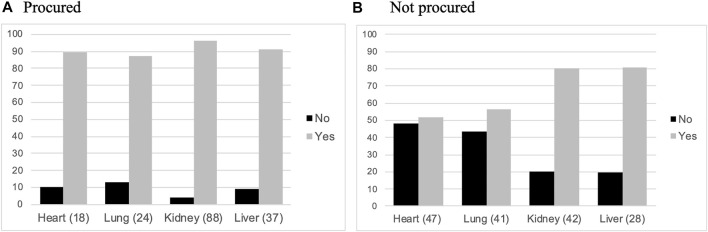
Perceived feasibility of organs for transplantation. Histograms representing relative proportions (%) of organs perceived as potentially feasible or not for transplant by the clinicians. Procured organs **(A)** and not procured organs **(B)**.

In the evaluation of organ suitability for transplant, 65 vignettes (second page) were transmitted to the clinicians and considered to be the denominator assessed (given on case rejected by all, after the first page assessment); they received a various proportion of their initial 50 cases that they had deemed feasible (A: 72%, B: 100%, C: 60%, D: 80%). It means that 13 vignettes were evaluated by two clinicians, 14 by one only and 38 of the 65 (58%) were evaluated by three clinicians, at this stage. Lung was considered as a whole, given the fact that on the 24 transplantation, a single lung was taken only in two cases. For the kidney, the total of the 88 (45 right + 43 left) were accounted for; both were procured in 39 and only one in 10 donors. For the organs, which were finally procured for transplantation, they predicted organ donation for the majority of the cases (Figure 1A), with less than 10% considered not feasible; they were more confident for kidneys, than for other organs (Supplementary Figure S1A). For organs, which were not procured, their predictions were more discrepant with the final outcome (Figure 1B), but in a lesser magnitude for hearts and lungs; they still thought that a majority of kidneys and livers could be procured for transplantation (Supplementary Figure S1B).

The reasons for final refusal of kidneys and livers by the OPO or the transplant team were multiples: mostly medical (e.g., past-medical history, suspect findings, compatibility with receiver, age, vascular anomalies, pathology findings per-op), consent changes or limitation by families, and no interest for the organ proposed.

## Discussion

The organ donation process is complex, resources-demanding and highly emotional, while the assessment of organ procurement feasibility is a challenging and dynamic process (13). In our study, we showed a high variability (moderate agreement) in a small sample of clinician’s assessment, despite the fact that they deemed the majority of their assessed cases feasible. At the stage of the initial short description of the potential donor, up to 36% of the potential donors could have been discarded, depending on who would have managed the case.

To initiate donation support process, critical care physicians must perceive a fair likelihood of reaching donation and at least provide one acceptable organ for transplantation. Critical care physicians conduct organ support and then expect potential recovery of organ failure (2, 11). In contrast, the OPO local coordinator collects specific clinical parameters (i.e., left ventricular ejection fraction, hepatic enzymes, creatinine level, oxygenation), at different time points and communicate them to the transplant teams. The latter have also their own perspectives: priorities, age of potential donors, matching, perceived quality of the organs, access to operating rooms, transplant team availability.

As illustrated by our study, despite a high sensitivity to predict a converted potential donor, opinions were far from unanimous. Potential barriers could not be collected in detail, but many causes could be mentioned: the clinician’s level of experience, the degree of confidence in the potential for maintenance or recovery of the organ function, the comfort in approaching family representatives, the perception of time needed for support and the access to eventual expertise in donor management (24, 25). Above all, we can also hypothesize that in an area as sensitive as organ donation, the perception of acceptability by colleagues and the institution is highly influenced by regional and institutional policies (26).

The perception of specific transplantable organs by the critical care physicians was high for the organ finally procured, but a more variable opinion was present for the organs not procured. An intriguing finding from our study is the discrepancy between clinician’s perception of kidney or liver transplant feasibility and reality. A large proportion of these two organs were finally not offered, considered or accepted for transplantation. Except for the presence of multiple organ failure in a few cases, we could not identify consistent items after the review of the medical chart, clarifying the barriers to donor conversion. As pointed out in an audit of the Spanish national registry donation process by external experts, a proportion of organs are sometimes excluded on the basis of medical contraindications deemed inappropriate (7). The central question is why?

Clinically, there are probably unclear boundaries on absolute and relative rules for eligibility, despite OPO efforts to generate criteria (27). In addition, this is a moving target with more and more borderline donors being considered. The latter generates the heterogeneity of potential organ donors, which despite critical care predetermined endpoints usually leading to more organ procured (20), complexifies the clinical assessment of eligibility. In the case of our vignette study, with the short clinical scenarios provided representing an initial snapshot, then a follow-up on organ investigations or support, the four clinicians could have missed the changes happening over time during the active organ support.

The acceptance of an organ by the transplant surgeon or team is usually conditional and the paradigm is skewed; a primarily accepted organ could subsequently be refused on the basis of new information, whereas a primary rejected organ is generally without appeal. It depends on the timing and the set of clinical information conveyed at that time; a critical care clinician with experience might be able to tailor the timing to allow organ recovery. Often, if the organs have already been refused and the offer is not renewed, enabling organ donation will require extra communication efforts. Historically, the principle of urgency for organ procurement was broadly applied. In France for example, organs are allocated with the condition that the transplant team proceeds within a 24-hour period. Although partly efficient, this approach excludes any possibility of giving a temporarily failing organ time to recover enough to be reconsidered.

In our study, granular arguments from the perspective of the OPO and transplant team were not available to enlighten their assessment of feasibility, other than generic decisions. For example, transplant nephrologist or hepatologist may decide, due to organ dimension, characteristics and various past-medical history of the donor, that the proposed organ is not suitable for a receiver (28, 29). A large proportion of organs deemed feasible by our external clinicians were finally not procured. Unlike overall critical care outcomes scores (30), organ function outcomes for donors are underdeveloped, despite the recent availability of decision’s algorithms for liver or kidney acceptance based on risks (31, 32).

In light of these observations, we believe that part of the reasons making the perceptions and outcomes so variable is the complexity of organ attribution system and the related processes. The literature showing the variability of acceptance in different centers supports this observation (17, 18). Moreover, transmission of clinical data as well as communication between the support and the transplant teams, are fragmented. The organ dispatching depends on what and how information is transmitted, often over the phone, and may lead to timely decisions that are not reassessed. Besides the biological/blood group matching of the proposed organs, the actual system depends on the variables related to the elements of allocations: 1) the timing; 2) local vs regional or national offer; 3) matching with borderline receiver (concept present on the donor’s side); 4) non-objective/non-systematic availability of donor medical information (verbally transmitted by OPO coordinators); 5) fragmentation of decisions, with stakeholders detached from the donor bedside. The current model of decision is based on urgency, with the sickest patient on the waiting list being considered first (33, 34). However, could the system consider offering refused organ to borderline receivers (or with less chance to go up the list)? The exact processes regarding decision-making are not always clearly defined or collected, thereby making difficult to precisely identify the present constrains.

To help us move forward, we would like to bring up in the discussion the example of decision’ process in oncology, typically involving multiple stakeholders. In this case, the best option for patients’ treatment and prognostication requires a multidisciplinary evaluation by an oncology board, including every decision-makers; the information is shared in a timely manner between a treating physician, a surgeon, an oncologist and radiotherapist, in order to decide for the best treatment applicable. It was demonstrated that these complex medical decisions, requiring the weight of medical information with the best option for a cancer treatment, can improve care (35, 36). In the case of potential donors, particularly those perceived as unlikely feasible, the medical information framework and the process of sharing could be better systematized, in order to avoid mislead decisions. The creation of a structured online canvas (similar to a registry of clinical data), where the patient’s characteristics, parameters and evolution overtime can be systematically documented (and automatically uploaded), could help to avoid subjectivity in the transmission of medical information. One can imagine that the critical care physician in charge of the patient, collaborating with the OPO coordinator, could feed real time information, specifications and also provide answers to questions from the transplantation team in a standardized manner. The development of algorithms testing the interaction between donors and recipients risk factors could help the teams and support a more objective system (37). We also propose the idea that the ultimate step would involve a session for more challenging cases in the format of a “donor board,” similar to an oncologic board meeting, in order to make consensual decisions and optimize the use of available organs. In addition, we believe that a dedicated donor supportive structure gives the possibility to allow time for evaluation, organ recovery and to enter a better window of opportunity where potential organs are optimized (19).

Our study has limitations, essentially regarding the small number of evaluators and the retrospective aspect of the design. It is nevertheless the only real-life data we could collect so far. In addition, we were not aware of the previous selection ratio of potential donors entering our system, adding potential bias in the number of borderline donors assessed. Furthermore, no emotional or cultural aspects were collected, regarding the approach to donor support. The four evaluators had however the possibility of assessing a high number of cases represented by real scenarios sufficient to test their agreement. A central aspect, that we did not consider for the analysis of our findings, is the difference in experience or expertise among the evaluators.

We acknowledge that the decision to cap the evaluation at 50 vignettes was based on our assumption that it would maximize the chance of response from the evaluators. Indeed, the variability of perception could have been lessened by the evaluation of the 66 potential donors by all evaluators. Another point is the possibility that the patient’s medical information extracted from the charts/database and transcribed to the vignettes lacked of informative precisions. First, the OPO was running its own inquiries (mostly through discussion with families) on the medical background of the potential donors, as well as the characteristics of potential receivers; secondly, the vignettes were built with summarized descriptions collected at the time of consent for organ donation; third, the new evolution of the potential donor medical condition, as well as the surgical assessment at the time of organ extraction was not reflected in the vignettes. Finally, the evaluations of the cases were done by physicians working in university hospitals, illustrating a limited representation of appreciation, since our province holds a large majority (>65%) of ICU beds in community hospitals. The opinion emanating from physicians outside of these centers could have provided a different variability of perceptions.

In conclusion, our study reveals that the support and assessment of a potential donor is a complex dynamic situation, involving different sources of medical information, with variability of perception in organ donation feasibility. To improve the overall system, we raise the possibility to standardize electronically the donor’s clinical/laboratory characteristics available to the transplant team, as well as the idea to test a “donor board” structure of decision. Further research, looking at the impact of such an approach in different healthcare system, is warranted.

## Data Availability

The original contributions presented in the study are included in the article/Supplementary Material, further inquiries can be directed to the corresponding author.

## Abbreviations

DBD, donation after brain death; DCD, Donation after Cardiac Death; OPO, Organ Procurement Organization’s; ORC, Organ Recovery Center.
