# Supplementary material for: The Suitability of Potential Organ Donors Using Real Case-Scenarios; Do we Need to Create a “Donor Board” Process for Donors Perceived as Unlikely Suitable?
Source: Transpl Int. 2022 Mar 10;35:10107. doi: 10.3389/ti.2022.10107 (PMC8944411; doi:10.3389/ti.2022.10107)
Supplement: Supplementary file 1 [file Presentation1.PDF]

Supplementary Figure 1

A. Perceived likelihood of transplant feasibility for procured organs

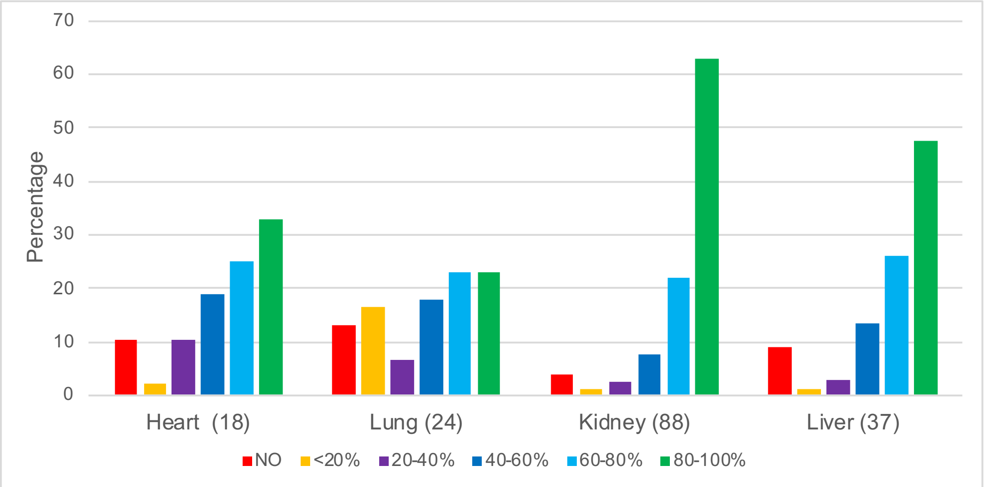

B. Perceived likelihood of transplant feasibility for the organs not procured

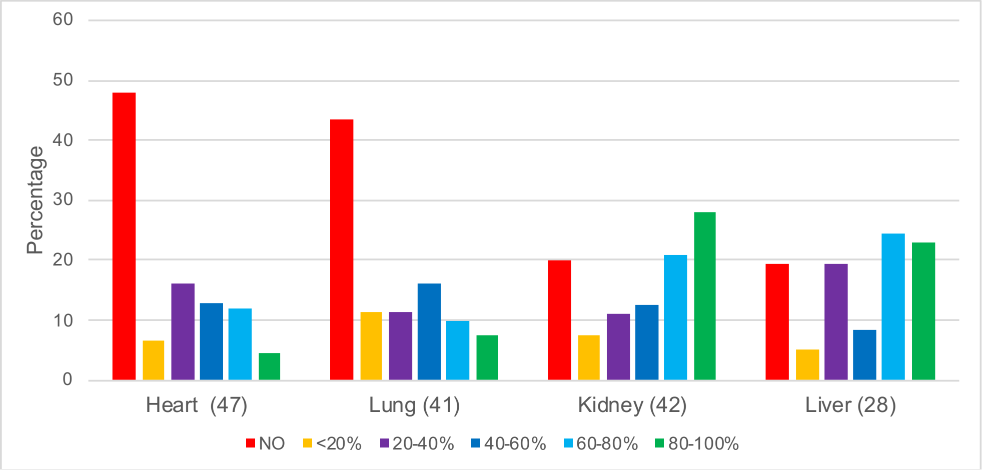

Histograms representing relative proportions (%) of organs perceived as potentially feasible or not for transplant, reported in the ordinal scale of likelihood given by the clinicians.
A. Finally procured organs. B. Not procured organs.
