# Supplementary material for: The Suitability of Potential Organ Donors Using Real Case-Scenarios; Do we Need to Create a “Donor Board” Process for Donors Perceived as Unlikely Suitable?
Source: Transpl Int. 2022 Mar 10;35:10107. doi: 10.3389/ti.2022.10107 (PMC8944411; doi:10.3389/ti.2022.10107)
Supplement: Supplementary file 2 [file Presentation2.PDF]

Example of Vignette document

The vignettes contained two pages: the first page had a short description of the potential donor at the time of admission. It contained the following information: age, body mass index (BMI), cause of brain death, past medical history, current disease history with new medical information (infection, positive serology, etc.), and physiologic parameters at the time of consent (arterial blood pressure, cardiac rhythm, presence of vasopressors, gasometry results, pulmonary x-rays, creatinine, liver enzymes, INR, temperature, lactic acid). The second page described the results of the investigations and physiologic parameters available for each organ, excluding the pancreas.

Clinical vignette part 1

Vignette #

1. Woman, 50 years old, BMI at 25, neurological death following intracranial hemorrhage

2. Habits: Alcohol consumption, Tabaco (20 Unit pack/year)

3. Past Medical History: dyslipidemia, arterial hypertension, type 2 diabetes, osteoporosis

1. Medical History: fall with facial trauma, hemiplegia, dysarthria
Neurological death in the context of hypertensive cranial hemorrhage
Significant hyperglycemia (11.8)
High blood pressure episode 190/95 to 200/115 with FC 130, sinus tachycardia

4. At the time of consent:
Vital signs: BP 120/80, Sinusal à 65, Lévo 0.05µg / kg / min, vasopressin 1.2U / h
Chest X-ray: Atelectasis at the two pulmonary bases
Blood gas: pH 7.14, PCO2 75, PaO2 250, Bic 24, PaO2/FiO2 250
Mean diuresis 60cc/h, creatinine52
AST 12, ALT 16
INR 1.10, no fever

Patient is candidate? Yes ☐
No ☐

If yes, feasibility Weak ☐
Moderate ☐
High ☐

Comments: _____

Clinical vignette part 2

Organ function and evolution

Heart
Echography: LVEF 65%, interventricular septum 6mm
Hemodynamic: TA 120/80, AF at 60/min, CVP 7; Norepinephrine 0.20µg/kg/min, vasopressin 1.2U/h

Non	<20%	20-40%	40-60%	60-80%	80-100%

Lungs
Blood gas: pH 7.40, PCO2 40, PaO2 330, Bic 20, PaO2/FiO2 330
Chest X-ray: right lower base atelectasis
Bronchoscopy: no secretions, normal

Non	<20%	20-40%	40-60%	60-80%	80-100%

Kidneys
Echography: left kidney 11cm, right kidney 10cm, no lesion
Diuresis 50mL/h,
Creatinine 56 (decreasing)

Non	<20%	20-40%	40-60%	60-80%	80-100%

Liver
Echography: no lesion, no steatosis
AST 14 (increasing)
ALT 18 (increasing)
INR 1.20 (increasing)

Non	<20%	20-40%	40-60%	60-80%	80-100%
